# Barriers to HPV self-sampling and cytology among low-income indigenous women in rural areas of a middle-income setting: a qualitative study

**DOI:** 10.1186/s12885-017-3723-5

**Published:** 2017-11-09

**Authors:** Betania Allen-Leigh, Patricia Uribe-Zúñiga, Leith León-Maldonado, Brandon J. Brown, Attila Lörincz, Jorge Salmeron, Eduardo Lazcano-Ponce

**Affiliations:** 1 Public Health Methods Department, Reproductive Health Division, Center for Population Health Research, National Institute of Public Health, Mexico City, Mexico; 2 National Center for the Prevention and Control of HIV and AIDS (CENSIDA), Mexico City, Mexico; 3 CONACYT, Center for Population Health Research, National Institute of Public Health, Cuernavaca, Morelos Mexico; 4 Center for Healthy Communities, Department of Social Medicine and Population Health, UCR School of Medicine, UC Irvine, Riverside, California, USA; 5 Center for Cancer Prevention, Wolfson Institute of Preventive Medicine, Queen Mary University of London, London, UK; 6 Center for Population Health Research, National Institute of Public Health, Av. Universidad #655, Colonia Sta. Ma. Ahuacatitlán, 62508 Cuernavaca, Morelos Mexico; 7 Epidemiology and Health Services Research Unit, Mexican Social Security Institute (IMSS), Cuernavaca, Morelos Mexico

**Keywords:** HPV test, Cytology, Self-sample, Cervical cancer, Barriers to detection, Middle-income nations, Qualitative methodology, Rural residence, Low-income, Underserved

## Abstract

**Background:**

Data is needed about barriers to self-collection of Human Papillomavirus (HPV) samples and cytology among low-income, disadvantaged women living in rural areas of lower-income countries as these women are at increased risk of cervical cancer mortality.

**Methods:**

Individual interviews (*n* = 29), focus groups (*n* = 7, 5–11 participants) and discussion groups (*n* = 2, 18–25 participants) were organized with women from three indigenous ethnic groups residing in rural areas in Mexico, after they were provided with free, self-sampled HPV tests. These groups are low-income, underserved by healthcare and have historically been on the receiving end of racism and social exclusion. Descriptive, qualitative content analysis was done, including two cycles of coding.

**Results:**

Interview and focus/discussion group data indicate women had limited understanding of HPV’s role in cervical cancer etiology. They identified HPV’s existence, that cytology detects cervical cancer, the need for regular testing and that cervical cancer is sexually transmitted. Organizational barriers to clinic-based cytology included irregular supplies of disposable speculums, distance to clinics and lack of clear communication by healthcare personnel. Women considered self-collected HPV-testing easy, less embarrassing and less painful than cytology, an opportunity for self-care and most felt they understood how to take a self-sample after a 20-min explanation. Some women feared hurting themselves when taking the self-sample or that they would take the sample incorrectly, which they worried would make the test useless. Attending HPV-testing in groups facilitated use by allowing women to discuss their doubts and fears before doing self-collection of the sample or to ask other women who were the first to do the self-sampling what the experience had been like (whether it hurt and how easy it was). Lack of indoor bathrooms was a barrier to doing HPV self-sampling at home, when those homes were resource-poor (one-room dwellings).

**Conclusions:**

Low-income, indigenous Mexican women residing in rural, underserved areas identified their need for cervical cancer screening but encountered multiple barriers to cytology-based screening. They found a number of advantages of HPV self-sampled tests. Employing self-collected HPV-testing instead of cytology could resolve some but not all gender-related, organizational or technical quality-of-care issues within cervical cancer detection and control programs.

**Electronic supplementary material:**

The online version of this article (10.1186/s12885-017-3723-5) contains supplementary material, which is available to authorized users.

## Background

Cervical cancer mortality has decreased in higher-income nations, given cervical cancer screening programs employing periodic cytology and follow-up of abnormal results [1]. Mortality rates have not declined in many middle- and lower-income countries [2, 3]. Lower screening coverage is associated with disparities which can constitute barriers to testing, including lower income and education, residence in rural or low human development index areas, belonging to indigenous groups or language and cultural barriers [4–7]. Beyond coverage, continued high cervical cancer mortality rates in lower-income countries are often due to inefficient screening programs [8].

Implementation of high-risk human papillomavirus (HPV) tests in screening programs could reduce mortality through more efficient early detection and treatment [8–11]. In large, middle-income countries such as India and Mexico, studies have shown clinician-administered and self-collected HPV testing to be more cost-effective than cytology alone [12]; when used in conjunction with cytology they identify more high-grade lesions which can progress to cancer [13]; and self-sampled HPV tests compare favorably with physician-sampling or cytology [11]. Also, studies have shown high acceptability of self-sampled HPV tests with increased participation by non-participating women [14, 15].

Given the advantages of HPV testing for lower-income countries with high cervical cancer mortality, low coverage and quality-control problems [8, 11, 14–16], where risk of dying of cervical cancer is often greater among women in rural and underserved communities [4, 6, 7], our objective was to study barriers to use of self-sampled HPV testing and cytology among low-income, indigenous women residing in rural areas of Mexico.

## Methods

The study protocol was reviewed and approved by the Research, Bio-security and Ethics Committees of the National Institute of Public Health of Mexico. The data from this study is not available for public consultation because the informed consent text which was read to participants included the statement: “Confidentiality: All the information you give us (during the interview or focus group) will be strictly confidential, meaning that it will only be used by the researchers of this project and will not be available for any other reason, except for selected quotations which may be included in research reports.”

### Sample and participant recruitment

We recruited women 20-years and older from three ethnic groups indigenous to Mexico, in three different states: Mam women in Chiapas, Nahuatl speaking women in Puebla and Huichol women in Jalisco. Mexico’s indigenous population generally has lower incomes, is more often illiterate or has not completed primary school and experiences multiple socioeconomic disparities. Their communities tend to be underserved by healthcare and have historically been on the receiving end of racism and social exclusion [17]. We included indigenous communities in the study since previous analyses had shown that women living in rural areas, in marginalized communities and in lesser developed parts of Mexico had a higher relative risk of dying of cervical cancer [4, 7, 18]. Also, using the limited data available, we were able to estimate that at the time of the study the cervical cancer mortality rate among women living in communities with higher proportions of indigenous population in Mexico was approximately 17 deaths per 100 thousand women 25 years and over, [19–21] as compared to a national rate of 9 deaths per 100 thousand women [22].

The study communities were rural and had low levels of human development, including limited access to quality healthcare. Most of the study communities had a small, rural health center (offering limited primary care), although two Nahuatl speaking communities in the Sierra Norte region of the state of Puebla did not have any health center in their community. Pap tests were performed at these clinics, although previous research indicates quality control at this type of rural health center is basically nonexistent [16]. The Mam communities in Chiapas and the Nahuatl speaking communities in Puebla all had access to a small hospital (secondary level care) in the capital of the municipality, while the Huichol communities in Jalisco did not. Distances to hospitals varied greatly, with some communities under 15 km from a hospital and others 50, 200 or 300 km from a hospital providing secondary level care. The communities generally had access to radio stations in their native languages and to radio and television in Spanish. Participants were low-income women from these communities and their fluency in Spanish ranged from proficient to non-existent.

This was a purposeful sample that sought to include cases that were expected to be rich in valuable information [23] given that these women are among those at greatest risk of mortality due to cervical cancer, [4, 7, 18] probably have greater barriers to early detection and treatment of cervical cancer and are those whom self-sampled HPV testing could potentially benefit most.

During 2007, upon arriving at each of the three regions studied, two female anthropologists recruited local female healthcare workers and bilingual residents to provide support with translation. Ten to fifteen days were spent with each ethnic group, with at least 3 days spent in each community or town. Some time was spent contacting community elders or women’s organizations; fieldworkers also travelled by foot through the communities with a megaphone (a method often used in rural communities in Mexico, to share news, offer services or goods for sale) promoting the study. Time was then spent going door-to-door to explain the study to women individually, and to invite them to either do the free HPV test at home (often rejected due to single-room homes with no privacy such as an indoor bathroom to perform the self-sampled test) or at a health clinic or other community center. Women agreeing to participate could opt to do only the free HPV test or to participate in an interview or focus group as well. Verbal informed consent was requested in the local language before participation*;* verbal consent was used instead of written consent because many of the women in the study population were illiterate.

At the health or community center, fieldworkers gave a 20–30 min educational session on HPV, cervical cancer and how to do self-collection for the hybrid capture-2 HPV test (specifying testing was free-of-charge with results in 6 weeks). Fieldworkers used illustrations of the self-sampled test being done by a woman, including an internal drawing of the woman’s vagina with the swab inserted, printed on large pieces of cardboard. All fieldworkers performed the self-sampled HPV test on themselves at the beginning of the fieldwork, so they had actual, personal experience of how the test was done, before explaining it to the participants. Women then performed the sample collection in a bathroom at home (if they had an indoor bathroom) or in the community center. Once placed in tubes with the buffer, samples were collected by the female fieldworkers and transported by them to the municipal capital and from there sent to a research laboratory for analysis. Results were provided in 5–6 weeks by the female fieldworkers, who returned to the study communities. Referrals for free cytology (for diagnostic confirmation) and treatment at the closest Ministry of Health clinic able to provide this care were provided to all women with a positive HPV test.

### Data collection

Nine focus or discussion groups (6 pre-testing, 3 post-testing) and 29 interviews were carried out, as were 503 self-collected hybrid capture-2 HPV tests (detailed information on data collection is available in a table [see Additional file 1]). Focus groups (audio-recorded) included 5–11 participants while discussion groups included 18–25 women (given the size of these groups, they could not be considered focus groups; also for this reason, they were not audio-recorded, as audibility would have been extremely limited). (Topics included in guides for pre- and post-testing focus and discussion groups and individual interviews are included in a table [see Additional file 2]). The same female anthropologist facilitated all focus groups, and another female anthropologist provided support and note taking. When interview and focus or discussion group sessions were translated, transcription was of the Spanish translation (for information on which interviews and group sessions were translated, see Additional file 1). Field-notes were taken during sessions since conditions were often not conducive to audio-recording.

### Data analysis

We sought commonalities in the qualitative data, which we segmented and distributed into categories in a two-stage coding process using descriptive categories for first-level coding and then pattern codes for second-level coding (for specific codes used in each stage, [see Additional file 3]). The pattern codes were based on selected literature on HPV testing [24–27], the anthropology of health and illness [28, 29] and gender theory [30, 31].

The data that was coded (in both stages) included transcriptions of the individual interviews and focus groups, and field notes taken during the discussion groups, which registered the statements made by participants as faithfully as possible. During both stages of coding, the transcriptions of audio-recordings and the field-notes taken when audio-recording was unfeasible, were read and re-read, and the codes were applied in order to identify and label data corresponding to the a priori categories, with some additional codes emerging from the ethnographic material. During the first stage of coding, the a priori codes were based on the topics of the interview/focus group guides while during the second stage, these codes were defined based on the literature review and theoretical framework. Next, segments (of the transcriptions or field notes) that had been classified within the categories (labeled with the codes) were grouped together and read through. For example, all the segments labeled with the specific code “Beliefs and knowledge about the cytology” were grouped together and read through. During this reading of the segments grouped by code or category, the first author interpreted, summarized and selected quotations for inclusion in the final write-up of the data [32].

## Results

Results are organized around the following themes: perceptions, beliefs and knowledge about cervical cancer and HPV; perceptions, knowledge and experiences related to cytology; barriers to cervical cancer screening using cytology; gender barriers and acceptability of the self-collected HPV test*;* perceived advantages of the self-sampled HPV test (for selected segments of the transcriptions or field-notes, grouped by topic or category, see Table 1, for a longer list of segments, [see Additional file 4]).

**Table 1:** Selected quotations from focus groups and individual interviews, study on HPV and cytology among rural, indigenous women in Mexico

Theme	Subtheme	Example quotation
Beliefs and knowledge about cervical cancer	Correct knowledge	“If you detect it in time, you can take that sickness out so it won’t progress any more and with a treatment you are fine.” (*Interview with a Nahua woman*)
	Incorrect knowledge	“Between these two sicknesses [HPV and cervical cancer] we’re in danger, we should go to the clinic or a doctor. If we feel pain in the womb, go to a doctor.” (*Focus group with Mam women*)
Perceptions of and knowledge about the Human Papillomavirus (HPV)	Correct knowledge	[Cervical cancer is caused] “by infections, because later they become like a tumor in the womb.” (*Interview with a Mam woman*) “There is a vaccine for human papillomavirus. I heard it on the radio, that there are vaccines.” (*Interview with Huichol woman*)
	Combined correct and incorrect knowledge	“Well, as far as I know, the virus is transmitted through sexual contact. Then this human papillomavirus, it begins without any warning. Then later it progresses, then the discomfort in our parts [genitals] begins and that’s when the discharge starts and it progresses to the cervix and when it gets to the cervix it goes into the uterus and that is when the doctor sends us for an operation.” (*Interview with a Nahua woman*)
Perceptions, knowledge and experiences related to cytology	Combined correct and incorrect knowledge	“Moderator: What is cervical cancer, what do you know about cervical cancer? Huichol woman 1: They say you can die of cancer, if you don’t detect it early. Moderator: And how do you detect it? Huichol woman 1: With the Papanicolaou, doing it periodically. Moderator: And what’s periodically? Huichol woman 1: Every three months. Moderator: Everyone, how often do you think you need to get a Papanicolaou? Huichol woman 2: Once a year. Huichol woman 3: Depends on how you feel, once a year or every two years, I get it every two years. Moderator: What do you mean, how you feel? Huichol woman 3: If you feel burning.” (*Focus group with Huichol women*)
Barriers to cervical cancer screening using cytology or the HPV self-sampled test	Pain and fear of unsterile equipment as a barrier	“[When they did the Papanicolaou test], maybe they did it wrong. I don’t know, but I felt something like a scrape. Then I thought maybe the equipment wasn’t disinfected. … I thought about it a lot before doing it again, because I was afraid.” (*Interview with a Nahua woman*)
	Not receiving test results as a barrier	“A lot of us say, and we’ve talked about this before, when we get a Papanicolaou, the results don’t arrive, and we don’t know what it is that’s going on. There we are with the doctor, asking why the results don’t arrive. … Whatever it [the result] is, they should give it to me.” (*Interview with a Mam woman*)
	Unequal gender relations as a barrier	“The first time I went to check myself, with the Papanicolaou tests, I had problems. I got beat-up. My husband hit me because he said I had gone to do things with the [male] doctor. When it wasn’t even a doctor who examined me, the [female] nurse examined me! She took the sample, but at home my husband didn’t believe that.” (*Interview with a Nahua woman*)
	Lack of gender-related barriers	“Because with all these [health education] talks they give us, women are more secure in themselves. So, we don’t really ask men for permission anymore, because it’s something that’s good for us.” (*Interview with a Huichol woman*) “I decided it [to perform the HPV test] myself, alone. I don’t ask anyone’s permission. … How am I going to ask him if he [her husband] wants it or not? It’s not for him, it’s for me.” (*Interview with a Nahua woman*)
Perceived advantages of the self-sampled HPV test	Less embarrassment	“This one [the HPV test] is good because it [cytology or the Papanicolaou test] really does embarrass you, not because your husband doesn’t want it or doesn’t let us, but because it’s embarrassing and because of embarrassment we don’t do it, and so this [self-sampled HPV test] is good for us.” (*Interview with a Huichol woman*) “because you do it yourself, since always, even if there is trust, you feel a little embarrassed to undress in front of someone else” (*Interview with a Nahua woman*)
	More comfortable	“Well, this one [the self-sampled HPV test] is better, because it is more comfortable to do it.” (*Interview with a Mam woman*)

### Perceptions, beliefs and knowledge about cervical cancer and human papillomavirus

All Nahua women interviewed knew of the existence of cervical cancer and many had heard of someone from a Nahua community dying from it. Many Nahua women mentioned their fear of cervical cancer, the need to have cytology in order to “detect it in time” (repeating phrases in Spanish from health education campaigns) and the existence of effective treatment. When asked about the causes of cervical cancer, Nahua women mentioned that “it comes from having lots of children”, “infection from your husband” or “because sometimes we’re with a lot of men”, although others said they had “forgotten” what causes it. Many Nahua women linked lack of hygiene with cervical cancer, said it was sexually transmitted and was asymptomatic during initial stages. In general, Nahua women (who also spoke more Spanish) had more correct and complete knowledge than the other two ethnic groups.

The Mam women mentioned both traditional and allopathic treatments for cervical cancer, seeming to prefer the former and fear the latter. One Mam woman seemed unconvinced of the efficacy of biomedical treatment for cervical cancer (perhaps also doubting its treatability). Another Mam woman spoke about “early detection” and “prevention” of cervical cancer, but later said she didn’t know what cervical cancer was, what caused it or if it could be cured. She repeated some of the information given the day before by the fieldwork team, but didn’t appear to have been convinced about the usefulness of testing. Misconceptions among the Mam women included that cervical cancer is caused by miscarriage or problems during childbirth and also lack of knowledge about the existence of an asymptomatic period during the development of cervical cancer.

The Huichol women spoke mainly of traditional treatments for cervical cancer, but generally considered it incurable or were doubtful of the efficacy of either traditional or allopathic treatments. However, two Huichol women expressed the desire to see if allopathic treatment was more effective and suggested combining the different types of treatment. In general, the Huichol women did not say they knew women who had been treated for or died of cervical cancer. The Huichol women mentioned the following as causes of cervical cancer: marrying very young, having many children, having vaginal infections, the last of which they said “come from not knowing who your man is with”.

Many of the Nahua women interviewed had received health education about HPV, knew HPV is sexually transmitted and identified a link between HPV and cervical cancer. Mam women generally had not heard of HPV although a few did say that a “virus” or “infection” causes cancer or tumors. Several Huichol women had heard of HPV, and generally spoke of beliefs and knowledge that combined traditional and scientific information. One Huichol woman said the following in response to a question asking what HPV is, “they are little, tiny animals. We don’t know what they are.” However, other women from this ethnic group stated that HPV causes cervical cancer and is sexually transmitted, and some knew an HPV vaccine exists.

### Perceptions, knowledge and experiences related to cytology

The Nahua women generally identified cytology as a diagnostic tool for detection, not confusing it with a treatment; some mentioned it detected simply illness or women’s illness, others said it detected cancer and some referred to cervical cancer specifically. Quite a few Nahua women mentioned that if cervical cancer were detected it was possible to treat it, through surgery, many mentioning hysterectomy and some describing less invasive surgical procedures. However, two Nahua women who had never had cytology in their lives (but did agree to have an HPV test), seemed to perceive the test as a way to prevent cancer or as a treatment instead of a diagnostic tool. Mam women generally knew what cytology was for, distinguishing it from prevention and mentioning that cervical cancer should be detected before it progresses to a more severe stage. While one Mam woman evidenced knowledge about a stage when cervical cancer lacks symptoms another woman showed a lack of this knowledge. In contrast, the Huichol women generally perceived cytology as a way to prevent cervical cancer or as a treatment for incipient cervical cancer, confusing detection with prevention or treatment. Although the concept of detection did not appear to be clearly understood, the Huichol women did link regular cytology use to prevention of morbidity (tumors, bleeding and the need for a hysterectomy) and of mortality due to cervical cancer. At the same time, they were unclear as to how often the test should be performed and about the need for periodic testing even in the absence of symptoms.

Women of all three ethnic groups reported limited previous gynecological healthcare. By far the most commonly mentioned type of care received was cytology, with little use of other types of care. Women generally used traditional birth attendants (midwives without formal training) for obstetric care, and giving birth in hospitals was only mentioned in a few cases. Some women who experienced complications were unable to access professional obstetric care, due to distance to the hospital and lack of an ambulance.

Across the three ethnic groups, many women reported undergoing cytology three-to-six times in their lives, generally in the recent past. However, one or two women in each ethnic group had never undergone cytology.

The participants’ had varied experiences of cytology collection: some women said healthcare personnel were respectful, explained the procedure, and that sample collection was slightly embarrassing but not painful. Others described cytology as extremely embarrassing and painful, and said healthcare personnel did not use disposable speculums, but “only washed them and reused them”, which was perceived as unhygienic.

Across the three ethnic groups, the women repeatedly mentioned that the healthcare system either took an inordinately long time to give them their cytology results, or did not provide them with their results unless they were positive. They coincided in that this was unacceptable and that it discouraged them from having regular cytology.

### Barriers to cervical cancer screening based on cytology

Women living in communities without a local health clinic mentioned distance (travel cost and time) to clinics as an important barrier to regular cytology use. In communities with a clinic, some women said that distance to hospitals was a barrier to treatment of cervical cancer if detected. Women with limited Spanish fluency mentioned a lack of healthcare personnel who spoke their language as something that made cytology difficult. Women also said they had difficulty understanding test results and treatment recommendations, which were explained in unfamiliar terms. Women who were illiterate mentioned that test results and treatment recommendations were sometimes given to them in writing, which meant they had to consult someone literate to understand them.

A significant barrier to regular cytology was the lack of a supply of disposable speculums at health clinics. Women said that sometimes when they arrived for a cytology appointment, they were told that the clinic had run out of disposable speculums and the woman would either have to buy one herself (this was considered prohibitively expensive) or return another day. Some women also said they did not have the economic resources to cover the cost of purchasing medicines to treat sexually transmitted infections detected during the pelvic exam for cytology. A few women complained of long waiting times for a cytology appointment and others said that women in the governmental poverty reduction program (now called *Prospera*, previously called *Oportunidades* among other names) were given priority over them, which meant they had to wait until those women had seen the physician, before they were seen.

### Gender barriers and acceptability of the self-collected HPV test

In the three ethnic groups there were gender issues that constituted barriers to previous cytology use or to acceptability of the self-collected HPV test. In all three ethnic groups there were some women who said they needed to “consult” their husband or “ask his permission” before deciding to have the HPV test or cytology. Among the Nahua women this was less common. When the self-collected HPV test was offered, one Nahua woman was undecided about whether to accept the test, and made up her mind based on her teenage daughter’s encouragement that she could make the decision on her own. In a number of cases when husbands were present in the home when the HPV test was offered to Nahua women, the men made comments that indicated they were supportive of or pleased with the idea of the women having the test. However, quite a few Mam women hid the fact that they were having the HPV test from their husbands. Some Mam women said they spoke with their husbands about getting cytology and that if he did not approve they would do it without his knowledge, hiding this fact from him. Although quite a few Huichol women said they needed to consult or discuss with their husband whether to have the HPV test, fewer said they needed his “permission” than among the Mam women. Huichol women mentioned discussing the decision with other family members, and appeared to want to make a group decision about the issue. However, other Huichol women said they decided individually what to do.

### Perceived advantages of the self-collected HPV test

Levels of acceptance of the HPV self-sample test were high, since among Mam women, 181 were offered the test and 15 refused; among Huichol women 135 were offered the test and 10 refused and among Nahuatl speaking women 223 were offered the test and 21 refused. We did not find patterns in terms of acceptability or perceived advantages of the self-collected HPV test among the women in terms of age or formal education, although since our study was qualitative the sample was small and with small numbers of women in each age group. Variability of education level was limited since most women had few years of formal education and were illiterate.

Nahua women said the self-collected HPV test was less embarrassing than cytology, given there was no need for undressing or a pelvic exam. Other Nahua women mentioned as advantages that they knew their own bodies better than healthcare personal and that the self-collected HPV test offered the opportunity for self-care. Huichol women also generally perceived the self-collected HPV test as less embarrassing and less painful than cytology. Nahua women concluded that a test they could perform themselves would tend to be less painful (since “we know each part of ourselves to do it, because … we know how to do it to ourselves better, we know how to do it.”) and in general more comfortable. Other women discussed the idea that the self-collected HPV test offered them the opportunity for self-care.

While the Nahua and also the Huichol women perceived the self-collected HPV test as “easy to use” and felt they could perform the sample collection correctly, the Mam women were divided, with some perceiving it to be easy to use and others having doubts about their ability to do the test correctly. The women who had doubts about how to do the self-collected test feared they might hurt themselves or that if the didn’t collect the sample correctly, the test would be useless.

Lack of indoor bathrooms was a barrier to doing HPV self-sampling at home, when those homes were resource-poor (those with one-room dwellings, with an outdoor latrine with no lighting). This led many women to opt for doing the self-sampled test at a location in the community, such as a health clinic (if one existed) or other community center. Related to this, an important advantage perceived by many of the indigenous women who participated in the study, which was not directly related to the HPV test being self-sampled, was that women attended the testing sessions in groups, instead of alone. For some women, this meant they could discuss their fears and doubts with other women before doing the test, which helped them decide to go through with it. Some women said they asked others after they had done their own self-collection what it had been like; when women who had gone before said the test was not painful and that they felt it was easy to do, others felt “encouraged” and decided to do the self-collection themselves.

## Discussion

Our study revealed a series of barriers to cervical cancer screening among low-income, indigenous women residing in underserved, rural communities. One type of barrier was incomplete knowledge: about HPV infection, cervical cancer etiology (existence of an asymptomatic period) and effective treatments. Facilitators related to knowledge included identifying early detection as important (even without specifically recognizing the need for testing in the absence of symptoms). Some women confused cytology (detection) with treatment, but this would not necessarily constitute a barrier to participation in screening. Organizational barriers to cytology included distance to clinics, use of comprehensible language by health personnel, long waiting times and delays in receiving test results (undermining motivation for future testing) and lack of disposable speculums at clinics. Specifically for the self-sampled HPV test, doubt about correctly collecting the sample or about if they might hurt themselves when doing the self-collection was a barrier for a few women. These issues in general indicate a need for additional counseling and health education; materials such as a manual and guidelines specifically on counseling for HPV testing (including the self-sampled option, and also including information about cervical cancer etiology, treatments and cytology) should be provided to healthcare workers, with advice on how to communicate this information in very simple terms [33]. The need for counseling and health education before and after HPV testing is done has been identified in other studies [34, 35]. The actual ability of the women participating in this study to carry out a self-sampled HPV test correctly is not in doubt, given that many other studies have shown that women can conduct self-collected samples which result in similar levels of accuracy as clinician-collected samples, in terms of detecting cervical intraepithelial cancer 2 or higher [36]. The issue studied here was whether women felt (or perceived) they would be able to do the self-sampling correctly; some had confidence in their ability while others did not, and participating as a group in the testing process was an important facilitator in terms of gaining confidence in being able to do the test correctly. Other studies have found lack of confidence in doing the test correctly among some, but not all, women; counselling and health education are usually recommended and have been found to increase confidence and acceptability [37–40]. We did not find patterns of acceptability of the self-sampled HPV test in terms of women’s age or formal education; other (quantitative) research has established that women’s age and education is not necessarily related to acceptability of HPV self sampled tests, [37] although is some populations age is a factor [41].

Differences between the three cultural groups studied were limited, and appear to be more a function of language proficiency than anything else. Nahua women more frequently identified that screening could detect cervical cancer in early stages when treatment would be effective. More Nahua and Mam women identified treatments for cervical cancer and believed they would be effective. Huichol women were more often unconvinced or unsure that cervical cancer can be detected in time and/or believed treatment to be ineffective. The similarities found may be due in part to the similar socio-economic situation these different ethic groups live in, including the tendency for their communities to be underserved by healthcare, receive racist treatment and be economically disadvantaged or low-income both at the individual and community levels.

Facilitators or ways in which self-sampled HPV tests could resolve barriers to cytology-based screening included experiencing less embarrassment or pain. Male partner opposition to a woman carrying out a cervical cancer test was not always resolved by the HPV test being self-sampled. This might indicate that when women give their male partner’s rejection of the pelvic exam as their reason for not having a Pap test, it may not be so simple. That is, there may be a combination of factors, including men’s attempts to control their female partner’s actions but also that the woman herself may not want to undergo a pelvic exam or another type of test, but finds her male partner’s rejection of it more socially acceptable than her own. The gender issues related to HPV and Pap test use are complex; while it appears clear that women’s empowerment will contribute to greater use of cervical cancer detection methods, including HPV testing, quality of care issues may be of equal, or perhaps greater, importance.

Also, women in this study saw attending screening in groups as an important facilitator. Attendance in groups allowed the women to discuss amongst themselves their doubts and fears before doing the self-collection of the sample or to ask women who were the first to do the self-sampling what the experience had been like (whether it hurt and how easy it was). Controlled trials of different group care models have shown that they reduce costs, achieve higher patient satisfaction, improve adherence to physician recommendations and in some cases produce improved health outcomes [42–45].

Other studies have found a variety of barriers to cervical cancer screening using cytology which are similar to those found in our study, such as lack of transportation, embarrassment, physical discomfort or pain during cytology, language barriers and lack of knowledge about screening [14, 15, 46]. Studies have generally found that women have high acceptance rates and positive attitudes towards self-sampled HPV tests; the most common disadvantage perceived by women (also found in our study) is being uncertain about performing the test correctly (fear of hurting themselves or taking the sample wrong and thereby making the test useless) [15, 26–34].

An important limitation of this study is that translation was done by local residents who had at most a secondary-level education (not professionals). This may have led to less in-depth data, omissions or modification of what women meant to express. However, since our research team found no other options for translation, we decided it was an acceptable alternative given the importance of obtaining data on this population, which is at greater risk of cervical cancer mortality. Given how recruitment was carried out, participants may have tended to be women who are more concerned about their health, have a more preventive mind-set, know more about the topics being studied and have a partner with more positive views about cytology and HPV testing. In addition, there may have been social desirability bias in the responses given; that is, women may have tended to say what they thought interviewers wanted to hear.

## Conclusions

HPV testing on self-collected samples has been shown to be an effective cervical cancer screening test for primary screening (with sensitivity comparable to clinician-collected samples, when a PCR-based test is used) [47, 48]. It could make high quality cervical cancer screening available to women with the greatest barriers: non-participant and underserved women, those with greater healthcare disparities and levels of poverty [4, 14, 15, 49, 50]. Incorporation of both self-sampled and healthcare personnel-collected HPV tests into cervical cancer screening programs in middle- and lower-income countries could be especially pertinent, given the burden of cervical cancer, lack of infrastructure and of adequate quality-control [13, 49]. The evidence suggests lower-income countries use HPV tests for cervical cancer screening, instead of cytology (which requires infrastructure that is unavailable and quality control which has been unsuccessful in these contexts) [8, 13, 16] or visual inspection (given its inaccuracy and potential for under- and over-treatment) [8, 42, 50]. Including a self-sampled HPV test for some women could make greater screening coverage possible while maintaining high quality, as well as resolving some of the barriers to cytology, as shown by our findings. Nevertheless, effective structures to guarantee adequate supplies, transport of samples, communication of results and in general the triage and follow-up system for women with positive results must be in place [13, 43, 49, 51]. In addition, innovative triage strategies as well as the combination of vaccination and screening for adult women (in addition to vaccinating girls) should be explored through studies to confirm cost-effectiveness and safety issues [51, 52]. In addition, culturally appropriate health education and counseling will remain important. Implementation studies and demonstration projects are needed in these areas to ensure the uptake of evidence-based detection methods for cervical cancer, and to guarantee that follow-up care (triage, including confirmatory diagnosis and treatment) is provided.

## Additional files

Additional file 1: Table listing data collection tools and participant characteristics-Allen-Leigh. List of data collection tools and participant characteristics, study on HPV and cytology among rural, indigenous women in Mexico. (DOCX 141 kb)

Additional file 2: Topics included in guides (data collection tools)-Allen-Leigh. Topics included in guides for pre- and post-testing focus and discussion groups and individual interviews, study on HPV and cytology among rural, indigenous women in Mexico. (DOCX 96 kb)

Additional file 3: Codes used for qualitative analysis-Allen-Leigh. Codes applied to qualitative data (field-notes taken during discussion groups, transcriptions of interviews and focus groups) in study on HPV and cytology among rural, indigenous women in Mexico. (DOCX 121 kb)

Additional file 4: Long table with quotations from qualitative data-Allen-Leigh. Table with quotations from focus groups and individual interviews organized by theme, study on HPV and cytology among rural, indigenous women in Mexico. (DOCX 123 kb)

## Abbreviations

HPV: Human Papillomavirus
