# Supplementary material for: Barriers to HPV self-sampling and cytology among low-income indigenous women in rural areas of a middle-income setting: a qualitative study
Source: BMC Cancer. 2017 Nov 9;17:734. doi: 10.1186/s12885-017-3723-5 (PMC5679364; doi:10.1186/s12885-017-3723-5)
Supplement: Supplementary file 1 — Table listing data collection tools and participant characteristics-Allen-Leigh. List of data collection tools and participant characteristics, study on HPV and cytology among rural, indigenous women in Mexico. (DOCX 141 kb) [file 12885_2017_3723_MOESM1_ESM.docx]

Additional File 1. List of data collection tools and participant characteristics, study on HPV and cytology among rural, indigenous women in Mexico.

|  | *Mam ethnic group, Chiapas* | *Huichol ethnic group, Jalisco* | *Nahuatl-speaking ethnic group, Puebla* |
| --- | --- | --- | --- |
| *Data collection activities* | 2 pre-testing focus groups (n=6 and 8)  1 post-testing discussion group (n=25)  10 interviews with women  166 self-collected HPV tests  15 non responses to (did not accept) HPV testing | 1 pre-testing focus groups (n=6)  1 pre-testing discussion group (n=18)  1 post-testing focus group (n=7)  9 interviews with women  135 self-collected HPV tests  10 non responses to (did not accept) HPV testing | 2 pre-testing focus groups (n=5 and 11)  1 post-testing focus group (n=7)  10 interviews with women  202 self-collected HPV tests  21 non responses to (did not accept) HPV testing |
| *Participants* | Married, cohabiting and single women 27-63 years (focus and discussion groups) and 25-62 years (interviews) | Married, cohabiting and single women 20-50 years (focus and discussion groups) and 27-50 years (interviews) | Married, cohabiting and single women 27-57 years (focus and discussion groups) and 28-74 years (interviews) |
| *Language characteristics* | All focus and groups and the interviews were carried out in Spanish | All focus and discussion groups and the interviews carried out in Huichol language, with translation during the sessions by a local bilingual woman | 1 focus group carried out in Spanish and a focus and a discussion group in Spanish and Nahuatl, with translation during the sessions by a local bilingual woman  9 interviews in Spanish and 1 in Nahuatl with translation during the interviews by a local bilingual woman |
| *Communities included* | 4 communities in the Motozintla municipality, located in the Western Sierra Madre region of Chiapas: Francisco I. Madero, Miguel Aleman, Rincon del Bosque, Santa Ana Pinabeto | 2 communities in the Mezquitic municipality, located in northern Jalisco: San Andres Cohamiata, San Miguel Huaixtita | 4 communities in the Cuetzálan municipality, located in the Sierra Norte region of Puebla: Zacatipan, Tecoltepec, Ayotzinapan, Tzinacapan |
| *Characteristics of communities* | Small communities (ranging from 200 to 750 inhabitants at 2000 Census)  Number of Mam speakers in Chiapas at the time of the study: 19,957  Mountainous region with altitudes ranging from around 1400 up to 2860 meters above sea level, with mesas and small valleys, a number of rivers, springs and lagoons, a semidry and warm climate, with limited rainfall during summer to fall. | Small communities (400-550 inhabitants at 2000 Census)  Number of Huichol speakers in Jalisco at the time of the study: 13,649  Mountainous region with altitudes ranging from 800 to 2000 meters with an average altitude of 1900 meters (the study communities are at 1950 and 1156 meters), steeply hilled areas where agriculture is carried out and forests with widespread deforestation, with small valleys, and a semidry, warm climate. | Relatively small communities (with 452, 719, 1214 or 2618 inhabitants at 2000 Census)  Number of Nahuatl speakers in the state of Puebla at the time of the study: 218,083  The Nahuatl ethnic group living in the northern part of Puebla is culturally distinct from the other two Nahuatl-speaking groups that live in the western and eastern parts of the state.  Mountainous region with steep slopes and a limited number of narrow valleys with streams and rivers; altitude generally ranging from 1500 to 2500 meters above sea level; frequent heavy rains and high levels of precipitation, large rivers and frequent landslides. |
| *Fieldwork team* | 2 female anthropologists  3 locally employed female nurses (provided support only with recruitment of study participants) | 2 female anthropologists  1 locally employed female nurse and 2 medical students working in the region (provided support only with recruitment of study participants)  1 bilingual indigenous woman (local resident who supported with translation) | 2 female anthropologists  3 locally employed female nurses (2 bilingual, 1 semi-bilingual --listening comprehension but limited spoken ability--, provided support only for recruitment)  2 bilingual indigenous women (local residents who supported with translation) |
| *Time in each community* | 15 days fieldwork, educational sessions lasted 50-75 minutes | 12 days fieldwork, educational sessions lasted 55-75 minutes | 10 days fieldwork, educational sessions lasted 45-60 minutes |
