# Supplementary material for: Barriers to HPV self-sampling and cytology among low-income indigenous women in rural areas of a middle-income setting: a qualitative study
Source: BMC Cancer. 2017 Nov 9;17:734. doi: 10.1186/s12885-017-3723-5 (PMC5679364; doi:10.1186/s12885-017-3723-5)
Supplement: Supplementary file 2 — Topics included in guides (data collection tools)-Allen-Leigh. Topics included in guides for pre- and post-testing focus and discussion groups and individual interviews, study on HPV and cytology among rural, indigenous women in Mexico. (DOCX 96 kb) [file 12885_2017_3723_MOESM2_ESM.docx]

Additional file 2. Topics included in guides for pre- and post-testing focus and discussion groups and individual interviews, study on HPV and cytology among rural, indigenous women in Mexico.

| **Interviews** | **Pre-testing focus or discussion groups** | **Post-testing focus or discussion groups** |
| --- | --- | --- |
| - Conceptions, representations and beliefs of health and illness in general and of cervical cancer specifically - Knowledge and beliefs about HPV, cytology (Papanicolaou) and HPV testing - Experiences of prenatal and obstetric care, family planning and cytology (Papanicolaou) - Gender issues related to healthcare use* | - How health, illness, cervical cancer and HPV are understood locally - Knowledge and beliefs about HPV, cytology (Papanicolaou) and HPV testing - Experiences of prenatal and obstetric care, family planning and cytology (Papanicolaou) - Gender issues related to healthcare use* - Community priorities in the area of sexual and reproductive health | - Presentation by study team of information about cervical cancer, HPV and HPV testing - Question and answer period - Women’s experience of the self-sampled HPV test - Women’s opinions on how self-sampled HPV testing should be provided (organizational issues) - Women’s expectations and needs about how HPV test results should be given to them - Community priorities in the area of sexual and reproductive health, especially focusing on cervical cancer detection |

*Gender issues were explored with questions about what women can or cannot do, what they should or should not do, and whether they need permission from their husbands, in terms of using healthcare in general, prenatal and obstetric care, family planning and cytology.
