# Supplementary material for: Barriers to HPV self-sampling and cytology among low-income indigenous women in rural areas of a middle-income setting: a qualitative study
Source: BMC Cancer. 2017 Nov 9;17:734. doi: 10.1186/s12885-017-3723-5 (PMC5679364; doi:10.1186/s12885-017-3723-5)
Supplement: Supplementary file 3 — Codes used for qualitative analysis-Allen-Leigh. Codes applied to qualitative data (field-notes taken during discussion groups, transcriptions of interviews and focus groups) in study on HPV and cytology among rural, indigenous women in Mexico. (DOCX 121 kb) [file 12885_2017_3723_MOESM3_ESM.docx]

Additional file 3. Codes applied to qualitative data (field-notes taken during discussion groups, transcriptions of interviews and focus groups) in study on HPV and cytology among rural, indigenous women in Mexico.

| 1. ***General, descriptive or first-round codes:*** applied to the data in the first round of coding as “exploratory, summarizing, sense-making” labels (Miles & Huberman 1994: p. 86). These codes were applied in a first step in order to begin to organize and understand the qualitative data. |
| --- |
| - 1. Concepts of health and illness   2. Gender issues and women’s rights   3. Cervical cancer   4. Cytology (Papanicolaou)   5. Sexually transmitted diseases   6. Human papillomavirus   7. Responses to the (self-collected) HPV test |
| 1. ***Specific, pattern or second-round codes:*** applied in the second round of analysis in order to “understand patterns, recurrences, plausible whys”. These were used as “explanatory or inferential codes, ones that identify an emergent theme, configuration or explanation. They pull together a lot of material into more meaningful and parsimonious units of analysis.” (Miles & Huberman 1994: p. 69). Applying these codes in a second step in the analysis allowed a systematic approach in order to better understand and interpret the qualitative data. |
| - 1. *Beliefs and knowledge related to health and illness (as elements of HPV test acceptability)*      1. Beliefs and knowledge about health and illness      2. Beliefs and knowledge about cervical cancer (the logic of how it works)      3. Beliefs and knowledge about the human papilloma virus      4. Beliefs and knowledge about the cytology      5. Place of cervical cancer in the women’s imaginary      6. Place of HPV in the women’s imaginary      7. Experiences with cervical cancer |
| - 1. *Organizational (healthcare) barriers and facilitators to HPV test acceptability*      1. Language barriers/facilitators      2. Cultural competence barriers/facilitators      3. Healthcare from a gender perspective      4. Knowledge about how the health care system works      5. Perceived healthcare accessibility (geographical, economic)      6. Previous experience of health care use (including obtaining a cytology, emphasis on organizational issues)      7. Gynecological experiences (pelvic exams, experience with vaginally inserted objects, mention of cytology focusing on the bodily experience) |
| - 1. *Gender-related barriers and facilitators to HPV test acceptability*      1. Gender roles (division of labor, housework and childcare assigned to women)      2. Gender identity (representations and discourses about women, self-concept and body-image issues related to gender)      3. Gender inequality (unequal gender relations)      4. Women’s agency (women’s ability to exercise power, especially over their own actions and bodies, to pursue their own goals and interests including use of early detection of cervical cancer) |

Miles MB, Huberman AM. Qualitative data analysis: An expanded sourcebook. Thousand Oaks: Sage, 1994.
