# Supplementary material for: Barriers to HPV self-sampling and cytology among low-income indigenous women in rural areas of a middle-income setting: a qualitative study
Source: BMC Cancer. 2017 Nov 9;17:734. doi: 10.1186/s12885-017-3723-5 (PMC5679364; doi:10.1186/s12885-017-3723-5)
Supplement: Supplementary file 4 — Long table with quotations from qualitative data-Allen-Leigh. Table with quotations from focus groups and individual interviews organized by theme, study on HPV and cytology among rural, indigenous women in Mexico. (DOCX 123 kb) [file 12885_2017_3723_MOESM4_ESM.docx]

Additional file 4. Table with quotations from focus groups and individual interviews organized by theme, study on HPV and cytology among rural, indigenous women in Mexico.

| **Beliefs and knowledge about cervical cancer** |
| --- |
| “If you detect it in time, you can take that sickness out so it won’t progress any more and with a treatment you are fine.” (*Interview with a Nahua woman*)  “Sometimes [we get it] because we don’t take care of ourselves, because we don’t bathe, and sometimes also it’s transmitted by the husband, sometimes he has a lot of women and then he comes with us and gives us this disease.” (*Interview with a Nahua woman*)  “She uses rattlesnake, she dries it and grinds it up. You eat it mixed with food. She also gives her patients Aloe Vera. She mixes it in a blender and gives the patient a glass of it, and she should drink it daily. You’ll see, for four, five days. Because the cancer, there’s a ball, it has the consistency of grains of sand. By the fourth day, the person, they start to look for it, it begins to fade away. By a month, they don’t have anything, with that plant.” (*Interview with a Mam woman*)  “Then they take out our womb. Once they take out the womb, there is a wound there. So, then it happens like with a tomato. If it is rotten in one part, and you cut that part off, and you put it away, and the next day you go and see, it’s rotten again, and you have to cut off another piece, and then it’s useless.” (*Interview with a Mam woman*)  “You treat it going with doctors. I’ve heard there are women that have it burned, and with that treatment they say they get well, but I really don’t know. And I’ve heard others say that when cancer of the womb is advanced what they do is take it [the womb] out.” (*Interview with a Mam woman*)  “Uterine cancer is from miscarriage, or as my friends say, many women risk having their babies alone, and sometimes pieces are left inside or there is a tear. That’s what cancer comes from.” (*Focus group with Mam women*)  “Between these two sicknesses [HPV and cervical cancer] we’re in danger, we should go to the clinic or a doctor. If we feel pain in the womb, go to a doctor.” (*Focus group with Mam women*)  “If you’re suffering from cancer or are in the process [of developing cancer], even if you go with a singer [healer] to be cured, you can’t. If they give you a home remedy or they give you something it’s just for a while but the sickness continues.” (*Interview with a Huichol woman*)  “Kupierrira [word in Huichol language used for cancer], which is fire to us, it can’t be cured. Our own singers [healers] who you go to, to be cured, they know that the sickness can’t be cured. They give you remedies. They sing for a night to see if they can scare the sickness off. But no, there is nobody who can cure it. You just feel better for a while, but it isn’t cured and then the person dies of it.” (*Interview with a Huichol woman*)  *On treatment of cervical cancer, Huichol women in a focus group commented that:* “They should give us medicine. The natural ones don’t cure you, they just ease the sickness.”  “[Treat it] with singers [healers] and pills, with both. I prefer both, maybe treating it like this doesn’t cure it, but if you go to a clinic, maybe they cure it with medicine.” |
| **Perceptions of and knowledge about the Human Papillomavirus (HPV)** |
| “Many men are on the other side [in the United States] and they are the ones who bring this virus. Once I was in a workshop where they told us that when the husband arrives, he should have a checkup so there won’t be problems, because if not, it spreads.” [*Another woman responded*.] “That’s what condoms are for, you have to use them [to prevent] the spread of infections. If not, if the husband is with one, two, three, four [women] and comes to me, he brings it to me. But if we protect ourselves, well, then nothing happens. But if you don’t, who knows.” (*Focus group with Nahua women*)  “Well, as far as I know, the virus is transmitted through sexual contact. Then this human papillomavirus, it begins without any warning. Then later it progresses, then the discomfort in our parts [genitals] begins and that’s when the discharge starts and it progresses to the cervix and when it gets to the cervix it goes into the uterus and that is when the doctor sends us for an operation.” (*Interview with a Nahua woman*)  *Cervical cancer is caused* “by infections, because later they become like a tumor in the womb.” (*Interview with a Mam woman*)  “When as a woman you have discharge, and it’s white, we believe that it is a disease that is called the tobacco disease. But it is created by the gods to be sent to that person. That is one of the beliefs that exist among us, about the tobacco disease. It is handed down by our ancestors. For us, for the elders, before, tobacco was sacred. If our great-grandfather planted a plant, and our grandfather planted, then the grandson also has to plant. If these beliefs aren’t followed women get this disease. This belief was…before really, because before I did believe this, in the tobacco disease. But now that they gave us talks, I think it is more like a vaginal infection. That’s where the cancer begins.” (*Interview with a Huichol woman*)  “The papillomavirus causes uterine cancer. Of course, the carrier is the man who transmits it sexually. Who knows where the man gets it? From being with a woman, that is what is known.” (*Interview with a Huichol woman*)  “There is a vaccine for human papillomavirus. I heard it on the radio, that there are vaccines.” (*Interview with a Huichol woman*) |
| **Perceptions, knowledge and experiences related to cytology** |
| *Examples of reasons women gave for seeking their most recent cytology (Papanicolaou) test*:  “To know how I am, to know how I am in my womb.” (*Interview with a Nahua woman*)  “Well because … we women can have this illness, such as cancer, cervical cancer, although breast cancer too, and you can die of it. So we have to prevent that with, well, you have to detect it, with the test.” (*Interview with a Nahua woman*)  *One Nahua woman said, in response to a question about how her last cytology went:* “Good, because now I know I don’t have an illness, so I feel calm. I feel good that I’m well. But if they told me I had something, an illness, I would be worried, but I would get treatment.” (*Interview with a Nahua woman*)  “You can be infected, you can be sick and you don’t realize you have it. Then you realize, you realize it when it is serious, when it is advanced, you know? So the Papanicolaou is to prevent these things, you know?” (*Interview with a Mam woman*)  “Mam woman 1: You get it [cervical cancer] because sometimes you don’t get your Papanicolaou test, and so you don’t know if you’re sick or not and … sometimes you find out you have cancer too late.  Moderator: Why do we get sick with cancer?  Mam woman 2: Because of infections. Because later they become like a tumor in the womb and you get it. Because sometimes you don’t get your Pap test and you don’t know if you’re sick or not, and sometimes it’s too late when you realize you have cancer.  Mam woman 3: Someone here in this community can get that illness. That’s why we have, that’s why thank God now we have, before we didn’t have the Papanicolaou that they test you with. Thank God we have that now, that test to see if we have it. That’s what the Papanicolaou is for, to know if you have cancer.” (*Focus group with Mam women*)  “Moderator: What is cervical cancer, what do you know about cervical cancer?  Huichol woman 1: They say you can die of cancer, if you don’t detect it early.  Moderator: And how do you detect it?  Huichol woman 1: With the Papanicolaou, doing it periodically.  Moderator: And what’s periodically?  Huichol woman 1: Every three months.  Moderator: Everyone, how often do you think you need to get a Papanicolaou?  Huichol woman 2: Once a year.  Huichol woman 3: Depends on how you feel, once a year or every two years, I get it every two years.  Moderator: What do you mean, how you feel?  Huichol woman 3: If you feel burning.” (*Focus group with Huichol women*) |
| **Barriers to cervical cancer screening using cytology or the HPV self-sampled test** |
| “When they did my Papanicolaou, they wanted to use a big device [speculum], and well, it looked big. They thought they needed a big speculum and they couldn’t put it in, they just hurt me. Then they had to change [to a smaller one] but they had already hurt me.” (*Interview with a Nahua woman*)  “But the second time I did it [the Papanicolaou test], I was scared to do it again, because they put a piece of glass inside me, a little tiny one, a little speculum.^[[1]](#footnote-1)^ But maybe they did it wrong. I don’t know, but I felt something like a scrape. Then I thought maybe the equipment wasn’t disinfected. … Then, this time, I thought about it a lot before doing it again, because I was afraid.” (*Interview with a Nahua woman*)  “A lot of us say, and we’ve talked about this before, when we get a Papanicolaou, the results don’t arrive, and we don’t know what it is that’s going on. There we are with the doctor, asking why the results don’t arrive. … Whatever it [the result] is, they should give it to me.” (*Interview with a Mam woman*)  *When offered the opportunity to do the self-sampled HPV test, Mam women responded with comments that indicate a gender-related barrier to screening:*  “I have to ask my husband.”  “I won’t do it [the HPV test], because my husband doesn’t like me doing that type of things. I use herbal treatments. Besides, my husband isn’t here to tell him.”  “Can I do it [the HPV test] now [at a neighbor’s house], and not at my home, because my husband is there?”  “Yes, it’s better if I do it here at [my neighbor’s] house. It’s just that my husband is at home and I don’t want him to know. I’m not sure about that, it’s better if I do it here.”  *A Nahua woman also spoke of a gender-related barrier to screening:*  “The first time I went to check myself, with the Papanicolaou tests, I had problems. I got beat-up. My husband hit me because he said I had gone to do things with the [male] doctor. When it wasn’t even a doctor who examined me, the [female] nurse examined me! She took the sample, but at home my husband didn’t believe that.” (*Interview with a Nahua woman*)  *However, other women commented that women should not need to ask men’s permission to do cytology or HPV testing:*  “The health educator said we can go to the doctor alone, even if the man doesn’t say so. We can do it on our own because we are taking care of ourselves.” (*Interview with a Huichol woman*)  “Because with all these [health education] talks they give us, women are more secure in themselves. So, we don’t really ask men for permission anymore, because it’s something that’s good for us.” (*Interview with a Huichol woman*)  “I decided it [to perform the HPV test] myself, alone. I don’t ask anyone’s permission. … How am I going to ask him if he [her husband] wants it or not? It’s not for him, it’s for me.” (*Interview with a Nahua woman*) |
| **Perceived advantages of the self-sampled HPV test** |
| *Nahua women said they preferred the self-collected HPV test to the cytology,* “because you do it yourself, since always, even if there is trust, you feel a little embarrassed to undress in front of someone else” (*Interview with a Nahua woman*) and “because we apply it ourselves alone. Well, here we have the custom of being embarrassed.” (*Interview with a Nahua woman*)  *Another Nahua woman said she preferred*: “The one you gave me, the papilloma [HPV test], is better for me because we do it ourselves. We know each part of ourselves to do it. Because … we know how to do it to ourselves better, we know how to do it.” (*Interview with a Nahua woman*)    “Well, this one [the self-sampled HPV test] is better, because it is more comfortable to do it.” (*Interview with a Mam woman*)  “I think it [the self-sampled HPV test] is safer for us. Well, because we have a little shyness, like with the doctors. I think it is a little less nerve-racking for us.” (*Interview with a Mam woman*)  “They [the fieldwork team] encouraged us, and we encouraged each other. Between us all, we talked and, me too. We heard there was no pain, that’s why I think it was better. Yes, we encouraged each other. When we are not alone it is easier, because the ones that never decide to do it [Pap testing], decided to [do the self-sampled HPV test].” (*Interview with a Mam woman*)  “They [the fieldworkers], encouraged us, and we encouraged each other. Between us all, we talked … We heard there was no pain, that’s why I think it was better. Yes, we encouraged each other. When we are not alone it is easier, because the ones that never decide to do it [Pap testing], decided to [do the HPV self-sampled test].” (*Interview with a Mam woman*)  “This one [the HPV test] is good because it [cytology or the Papanicolaou test] really does embarrass you, not because your husband doesn’t want it or doesn’t let us, but because it’s embarrassing and because of embarrassment we don’t do it, and so this [self-sampled HPV test] is good for us.” (*Interview with a Huichol woman*)  “It’s [the self-sampled HPV test] more comfortable, because sometimes, because of shame … we don’t do it [the Pap test]” (*Interview with a Huichol woman*)  “Well, I did it myself, alone, so this one [the self-sampled HPV test] is better for me.” (*Interview with a Huichol woman*) |

1. A commonly used term for speculum in Spanish, “espejo”, is the same word for mirror, which may have contributed to this woman’s perception that a piece of glass was inserted. Or it could also have been related to the fact that disposable speculums are made of clear plastic. [↑](#footnote-ref-1)
